# Post-Lobectomy Pleural Aspergillosis with Bronchopleural Fistula in a Patient with Metastatic Synovial Sarcoma of the Lung: A Case Report

**DOI:** 10.3390/jcm15051734

**Published:** 2026-02-25

**Authors:** Angeliki Katsarou, Konstantinos Thomas, Ioannis Grigoropoulos, Anastasios Kyriazoglou, Elias Santaitidis, Periklis Tomos, Wiktoria Skórka, Magdalena Mnichowska-Polanowska, Małgorzata Edyta Wojtyś, Konstantinos Kostopanagiotou

**Affiliations:** 1 4th Department of Internal Medicine, National and Kapodistrian University of Athens School of Medicine, Attikon University General Hospital, 12462 Chaidari, Greece; 2 2nd Propaedeutic Department of Internal Medicine, National and Kapodistrian University of Athens School of Medicine, Attikon University General Hospital, 12462 Chaidari, Greece; 3 Thoracic Surgery Department, National and Kapodistrian University of Athens School of Medicine, Attikon University General Hospital, 12462 Chaidari, Greecekostop@hotmail.co.uk (K.K.); 4 Student Scientific Club of Department of Thoracic Surgery and Transplantation, Pomeranian Medical University in Szczecin, 70-204 Szczecin, Poland; 5 Department of Microbiology Immunology and Laboratory Medicine, Pomeranian Medical University in Szczecin, Powstańców Wielkopolskich 72, 70-111 Szczecin, Poland; 6 Department of Thoracic Surgery and Transplantation, Pomeranian Medical University in Szczecin, Alfreda Sokołowskiego 11, 70-891 Szczecin, Poland

**Keywords:** pleural aspergillosis, antifungals, topical therapy, bronchopleural fistula

## Abstract

In clinical practice, healthcare providers encounter a rising incidence of aspergillosis, which significantly affects morbidity and mortality in vulnerable patients. Over the past few decades, molds have increasingly affected patients with underlying pleuropulmonary, hematological, or oncological diseases undergoing cytotoxic treatment or immunosuppression, leading to impaired cell-mediated immunity and an increased risk of postoperative complications. Although the spectrum of *Aspergillus* infection is variable, ranging from allergic to chronic, invasive manifestation, pleural involvement is rarely reported. Pleural aspergillosis is an extrapulmonary manifestation of invasive aspergillosis, associated with thoracic surgical procedures and with a bronchopleural fistula, not necessarily combined with pulmonary aspergillosis. An elective or emergency thoracic surgery in immunocompromised patients increases the risk of postoperative infectious complications. Herein, we report a case of isolated postoperative pleural aspergillosis in a 28-year-old immunocompromised man with metastatic synovial sarcoma in the lungs, who underwent pleurodesis for pneumothorax, lobectomy for lung metastasis, and subsequently required decortication and thoracoplasty to achieve effective control of infection. To address this, the patient responded well to aggressive surgical debridement along with both systemic and intrapleural antifungal agent instillation. The essential in vitro diagnostics, including microscopy, microbiological culture and histopathological examination, both from necrotic pleural specimens, detected *Aspergillus fumigatus*, a global priority species of invasive aspergillosis. Postoperative aspergillosis with pleural involvement and bronchopleural fistula, in immunocompromised patients with sarcoma, is rarely reported, requiring a combination of surgical approach and optimized antifungal treatment regimens. The current knowledge on pleural aspergillosis management remains limited, and highlights the need for case reporting to refine expertise.

## 1. Introduction

The prevalence of aspergillosis increasingly affects patients with underlying pleuropulmonary, hematological, or oncological diseases undergoing cytotoxic treatment or immunosuppression, consequently leading to impaired cell-mediated immunity. More than 2 million at-risk individuals with underlying lung diseases develop invasive aspergillosis annually, with mortality exceeding 85% in some cases [1] and with an approximately 25% risk of systemic fungal dissemination [2]. This disease constitutes 27% of U.S. healthcare costs related to all fungal infection, representing a notable economic burden [2]. Aspergillosis is manifested as allergic bronchopulmonary aspergillosis (ABPA), chronic pulmonary aspergillosis (CPA) and invasive pulmonary aspergillosis (IPA) [2,3]. The severity and duration of neutropenia drive the risk of invasive aspergillosis, and this clinical condition is a major cause of morbidity and mortality in immunocompromised patients [4].

Pleural aspergillosis is a rare extrapulmonary manifestation of invasive aspergillosis, associated with thoracic surgical procedures and with poor prognosis when complicated with a bronchopleural fistula [5]. Pleural aspergillosis may present as an isolated form or in association with pulmonary aspergillosis. It occurs primarily in individuals with aggressive immunosuppression, but also in those without immunosuppression [6,7]. Pleural aspergillosis was first described by Cleland in 1847. It is unlikely to be related to allergic bronchopulmonary aspergillosis (ABPA) and it does not have to be associated with invasive pulmonary aspergillosis (IPA) [8].

Pleural aspergillosis occurs most commonly in the setting of pleural empyema or a bronchopleural fistula or pleurocutaneous communication, where continuous air leak into the pleural cavity and necrotic exudate provide favorable conditions for fungal growth [8]. Extensive necrosis or fibrosis of pleura and chest wall were found to be associated with *Aspergillus* infection.

*Aspergillus fumigatus*, as a major cause of IA, is widespread in the environment, making human exposure via spore inhalation or ingestion inevitable [3]. The guidelines of the Infectious Diseases Society of America (IDSA) strongly recommend reducing mold exposure to patients at high risk for IA [2]. A proven postoperative case of invasive aspergillosis should be sufficient to consider previous patients’ colonization or initiate epidemiological investigations to prevent postoperative complications [9]. The unique and extremely rare form of invasive aspergillosis involves the pleural cavity and can result in bronchopleural fistula. Pleural aspergillosis was diagnosed both in an immunocompromised 28-year-old man, presented by the authors of this report, and in an immunocompetent 48-year-old man, emphasizing that mold colonization of the pleural cavity could be secondary to surgery [10]. Post-lobectomy pleural aspergillosis can be fatal unless diagnosed and treated promptly [5,9]. The proper diagnosis of this requires adherence to evidence-based guidelines. Reference standards for diagnosing cases of pleural aspergillosis involve use of invasive procedures to obtain pleural specimens for culture and histopathological examination. The reliable diagnosis is based on less-specific clinical data and radiological findings as well as evidence of laboratory-confirmed fungal growth and histopathological examination. Pleural aspergillosis may represent either colonization or invasive disease, depending on evidence of tissue invasion. Research definitions for the proven, probable and possible invasive fungal infection, recommended by EORTC/IFICG/MSG, are helpful in clinical-decision making, particularly for immunocompromised patients with cancer [6]. Guidelines for diagnosis of IPA were recently published by a multidisciplinary group of experts representing the American Thoracic Society, whereas there is a lack of such guidelines for pleural aspergillosis [11,12]. Currently, IA is identified and diagnosed in non-neutropenic individuals treated with novel agents (e.g., biologicals, kinase inhibitors, chimeric antigen receptor T cells) [2,13,14].

The treatment of pleural aspergillosis, mainly in immunocompromised patients, remains challenging because knowledge about its management is limited. Monotherapy with voriconazole is considered as a primary treatment with surgical resection [2,15]. Voriconazole exhibits activity against all *Aspergillus* species, excellent bioavailability, potential therapeutic drug monitoring, and good penetration into the pleural space [16]. A promising option is isavuconazole, an extended-spectrum triazole, which has been approved in 2015 by the Food and Drug Administration (FDA) and the European Medicines Agency (EMA) for the treatment of invasive pulmonary infections [17].

Pleural aspergillosis has a relatively rapid progression and its management requires a combination of systemic antifungals with expeditious surgical debridement. Echinocandins were combined with other antifungals in patients with severe or refractory IPA.

Here, we report the case of a young male with metastatic synovial sarcoma who received multiple thoracic procedures of multidisciplinary treatment and developed extensive pleural aspergillosis early in the postoperative period, requiring thoracoplasty and systemic antifungal therapy combined with intrapleural instillation of antifungals. We highlight the necessity for a multidisciplinary approach for oncological surgery patients with soft tissue tumors whose infectious complications can be challenging concerning both diagnosis and management.

## 2. Case Presentation

**Figure 1:**
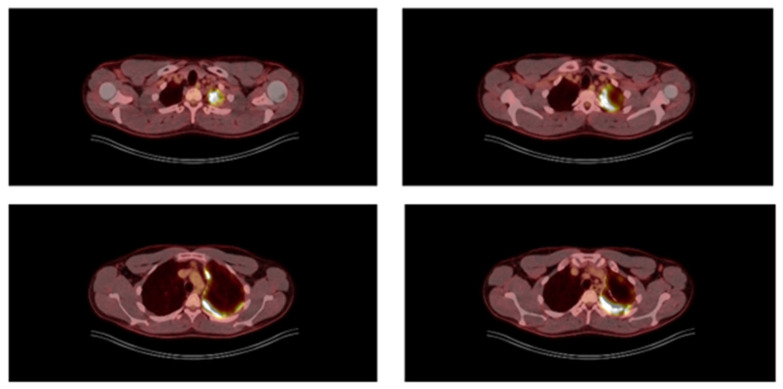
The preoperative staging ^18^FDG PET/CT scan aimed at detection of mediastinal lymphadenopathy and extra-thoracic metastatic sites. There were no such findings but the previously pleurodesis (left hemithorax) was pathologically avid even one year post talcage. This was erroneously attributed to the pleurodesis procedure but was in fact misdiagnosed pleural aspergillosis and was even unrecognized during the metastatectomy procedure.

A 28-year-old male diagnosed with synovial sarcoma of the upper extremity received three cycles of doxorubicin with ifosfamide plus 25 cycles of radiotherapy on the forearm before resection. Resection was successful with R0 margins, and histology confirmed synovial sarcoma. He was followed up for 3 years until he developed the first sign of metastatic disease in the left lung, manifesting as spontaneous pneumothorax. This was addressed by thoracoscopic (VATS) lingulectomy and talc poudrage, during which diffuse pleural deposits and a solitary parenchymal nodule were discovered. Upon resection, it proved to be metastatic. The patient recovered well without signs of infection and was discharged 4 days post-thoracoscopy. He received five cycles of doxorubicin and ifosfamide and was followed up for 1 year until computed tomography (CT) detected bilateral lung metastases in the form of three parenchymal nodules. On the right side, a 2 cm nodule in the upper lobe was treated by percutaneous image-guided thermal ablation (IGTA) in the interventional radiology suite. On the left side, there were two separate pure intraparenchymal metastatic lesions in the lower lobe (3 cm and 6 cm) not amenable to image-guided thermal ablation or wedge resection. Lobectomy was proposed by the multidisciplinary oncology meeting after staging and preoperative workup. The staging with ^18^FDG PET/CT scan was avid for all three pulmonary metastases, with an SUV_max_ of 10, and extensively avid for the left pleural surface, compatible with the previous talc pleurodesis 1 year prior. It was negative for any extra-thoracic disease. No lymphadenopathy (intra- or extra-thoracic) was detected (Figure 1).

Before lobectomy, the patient completed cardiopulmonary exercise testing with an estimated VO2max of 16.5 mL/kg/min, which is suboptimal for a young non-smoking adult.

A left thoracotomy and lower lobectomy with lymphatic dissection under single lung ventilation was completed after expeditious symphysiolysis due to previous pleurodesis. The postoperative recovery was uneventful with early mobilization and chest drain removal by day 8. On postoperative day 9, the patient began deteriorating by becoming febrile and showing significant elevation of laboratory inflammatory markers [white blood cell count, 24.300/μL; platelets, 1.165.000/μL; C-reactive protein, 174 mg/L (normal range: 0–6 mg/L)].

**Figure 2:**
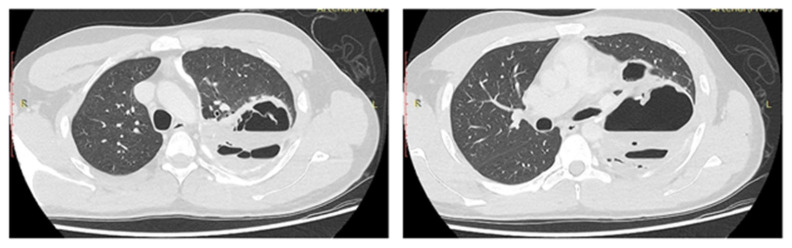
Computed tomography with intravenous contrast during the third postoperative week after redo left thoracotomy and lower lobectomy. The imaging was ordered to investigate fever and leukocytosis. Trapped lung (upper lobe) with air–fluid level and septae confirmed postoperative complex pleural empyema. This type of empyema is unlikely to respond to percutaneous drainage and requires revision surgery for debridement, decortication, and possibly thoracoplastic procedures (e.g., Eloesser flap).

His evaluation by the infectious diseases team did not reveal potential sites of infection except from the recent thoracotomy. Thus, a chest CT was performed in the beginning of the third week which showed a collection of increased density fluid and air in the left hemithorax, consistent with complex pleural empyema (Figure 2).

Redo thoracotomy was indicated to prevent sepsis. Intraoperatively, gangrenous lung tissue and peripheral bronchopleural communication were detected. Extensive debridement was performed across the pleural surfaces, removing necrotic material with infiltration characteristics not common to the usual bacterial pleural empyemas. The bronchopleural fistula (BPF), manifesting as free air bubbles on lung expansion, was repaired with direct parenchymal suturing. The remaining left upper lobe remained trapped under thick visceral pleura, a result of the previous pleurodesis; thus, decortication was unsuccessful. The under-expanded upper lobe was insufficient to fill the pleural cavity, with the remaining empty pleural cavity predisposing to empyema recurrence. For these complex pleural empyema cases with trapped lung, decortication combined with thoracoplasty is indicated. The fundamental thoracoplastic procedure is window thoracostomy or Eloesser flap, which is deemed mandatory for regular irrigation and debridement.

**Figure 3:**
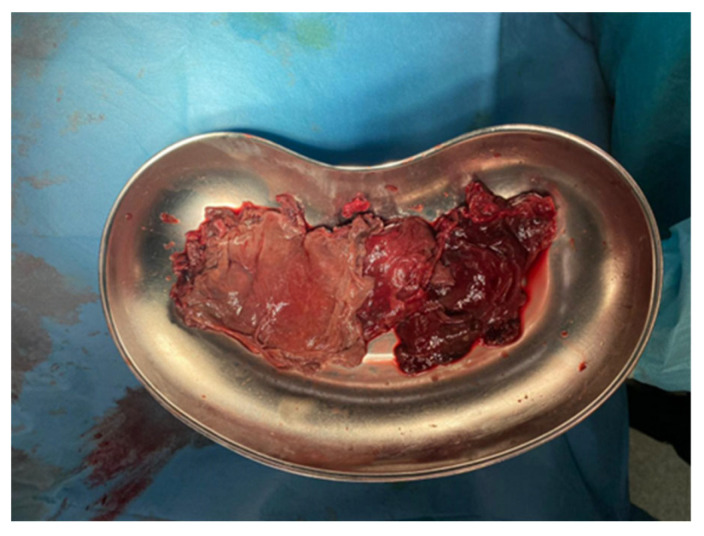
A significant quantity of necrotic tissue was removed during thoracotomy for debridement due to persistent fever and increased laboratory markers of inflammation. Bacterial pleural empyema does not manifest this way, and fungal infection should be suspected. Histopathological examination revealed hyphae, and tissue culture found *Aspergillus fumigatus*.

From pleural samples and necrotic masses obtained intraoperatively, *Aspergillus fumigatus* susceptible to voriconazole was cultured, while histopathological examination revealed PAS-positive septate hyphae with acute-angle branching, establishing the definitive diagnosis of proven aspergillosis (Figure 3).

At the time of diagnosis, serum galactomannan (sGM or GM) was undetectable. However, serum galactomannan may be negative in extrapulmonary or localized forms of aspergillosis, limiting its diagnostic utility in pleural disease [3,18].

**Figure 4:**
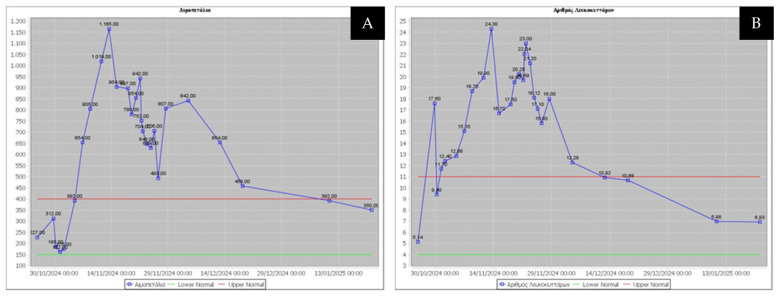
Thrombocytosis (**A**) and leukocytosis (**B**) were observed with a steep increase after the second postoperative week, indicating a developing infection that was probably related to the thoracic procedure. A thorough physical examination, cultures, and CT imaging are mandatory to reveal the source of infection. For patients receiving systemic chemotherapy, these findings alert us to oncoming sepsis. Once the source is recognized, surgical exploration should be encouraged. One week after the thoracoplasty procedure, there was a steep decline in inflammatory cell counts.

The patient was classified as a case of proven aspergillosis based on the EORTC/MSG mycological criteria used to diagnose IPA [13]. The finding of molds prompted a re-interpretation of the ^18^F-FDG PET/CT imaging, as the pleural thickening initially considered a pleurodesis-related inflammatory reaction was subsequently suspected to be a striking manifestation of pleural aspergillosis. Clinical and laboratory evaluations conducted one day after surgery demonstrated an immediate respiratory improvement following surgical source control, with resolution of fever and a significant decrease in inflammatory parameters (white blood cells, platelets, and C-reactive protein) (Figure 4A,B).

Systemic antifungal therapy was initiated following isolation of *A. fumigatus* from the pleural samples. Specifically, oral isavuconazole—instead of the first treatment choice, voriconazole—was administered to avoid potential drug interactions and concerns for enhanced toxicity of the upcoming chemotherapy with ifosfamide and doxorubicin. Pleural aspergillosis management is not standardized; therefore, after reviewing the relevant documented individual case data, daily intrapleural instillation of 25 mg liposomal amphotericin B (diluted in a 5% glucose solution), in the form of gauze pads impregnated with antifungal agent, was implemented through the open-window thoracostomy as adjunctive therapy.

The thoracostomy window gradually decreased in width and depth once cultures became negative for *Aspergillus* growth. The patient was finally discharged afebrile after 25 days of hospital stay. No wound healing complications occurred and partial lung expansion allowed unrestricted breathing.

## 3. Discussion

A patient undergoing thoracic procedures is at risk of developing postoperative infectious complications. The predisposing factors for pleural aspergillosis indicated by Chung et al. and observed in the presented case were bronchopleural fistula, pleural drainage, and lung resection, excluding pre-existing pulmonary tuberculosis [8]. The prevalence of aspergillosis has steadily increased over the years along with an increase in the proportion of patients with underlying immunosuppression [3]. The spectrum of patients at risk has evolved from the traditional profile of the neutropenic patient after intensive antileukemic chemotherapy or hematopoietic cell transplantation. A variety of risk factors, including structural abnormalities of the lung, cancer, or severe lung infection (e.g., viral, in particular by influenza or SARS-CoV-2) are now recognized as a predisposing background for mold colonization and, in turn, infection [19,20]. Colonization *per se* is not synonymous with infection; evidence supports *Aspergillus* colonization in the lungs of healthy subjects, with its DNA found in BAL in 37%, as well as in patients with chronic obstructive pulmonary disease (COPD), with positive cultures found in ~30% [13]. However, this is a prerequisite for the development of invasive infection [13].

Lung involvement in *Aspergillus* infections may vary from aspergilloma within a cavitary lesion to IPA, CPA, and ABPA [9,21,22]. Pleural involvement remains a rare complication of aspergillosis and is the result of *Aspergillus* dissemination from the lung parenchyma, bronchi, chest wall, or surgical site, or rarely, hematogenously [5], and it is frequently accompanied by the presence of BPF [21]. BPF is among the post-surgical complications of pulmonary aspergillosis [22]. Lobectomy itself comprises a risk for BPF (0.2–3%), with high mortality rates of 25% to 71% [23]. Risk factors for BPF include pre-, peri-, and postoperative events, comorbidities (COPD, diabetes), treatment (chemotherapy, radiotherapy, corticosteroids), and impaired nutrition [23,24].

**Figure 5:**
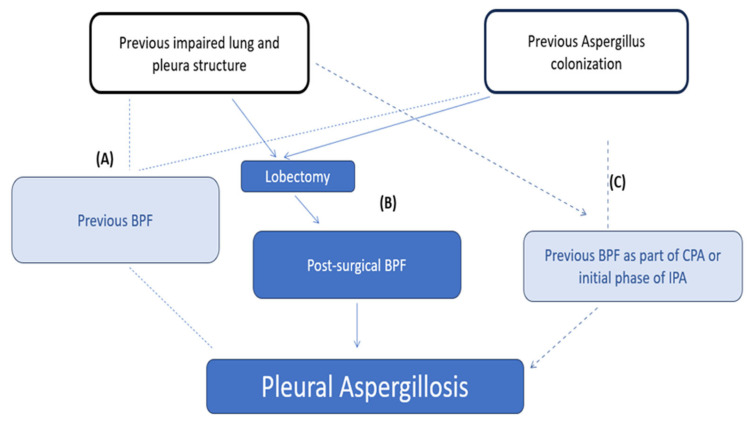
Three scenarios for the development of pleural aspergillosis in the patient. BPF: bronchopleural fistula; CPA: chronic pulmonary aspergillosis; IPA: invasive pulmonary aspergillosis. (**A**) Previous infection and/or damage leading to BPF. (**B**) Previous infection and/or damage leading to BPF post-surgery. (**C**) Continuation of infection.

Our patient had been previously subjected to ablation and chemotherapy and was mechanically ventilated postoperatively. According to a case series including 13 patients diagnosed with *Aspergillus* pleuritis, 10 of them had BPF; interestingly, 8 of 10 had a previous lobectomy, highlighting post-lobectomy BPF as a potential cause of pleural aspergillosis. In all but one patient, there was a concomitant bacterial infection; this contrasts with our case, where no other pathogen was identified in specimen cultures. In another case series of 18 adults with underlying pulmonary disease (e.g., COPD, emphysema, mycobacterial infection, pneumothorax, pleurodesis, lung cancer) who were diagnosed with CPA, pleural involvement with remarkable pleural thickening was found in 12 patients, but it evolved to BPF in only 1 patient; however, 3 out of 4 patients operated on as part of infection management developed pleural aspergillosis following the surgery [25]. In our patient, whether the BPF was a sign of primary infection developing during the course of CPA or early IPA, or a consequence of surgical site infection in a patient already colonized by the mold, remains unknown (Figure 5).

It is worth mentioning that fungal hyphae were present in the pleural cavity but not identified in the lung parenchyma. A. fumigatus may gain direct access to the pleural cavity as a result of thoracic surgery, pleural drainage or broncho-pleural fistula formation, finding favorable conditions for its growth. The residual pleural space (post-resection cavity) containing air after lobectomy, continuous air supply to the pleural cavity after chest tube drainage performed due to pneumothorax, or continuous air leak into pleural cavity due to BPF formation create favorable conditions for potential *Aspergillus* colonization. Fungal colonization associated with thoracoplasty procedures and/or communication between the environment, airways and the pleural space may increase the risk of dissemination of *Aspergillus* into the pleural cavity [26].

Postoperative necrotic masses with residual clots, fibrin deposits, cellular debris, and pleural exudate rich in proteins can provide a substrate for the growth of molds. The impaired healing in this patient receiving immunosuppressive therapy could have caused the persistence of necrotic deposits, and lack of proper mesothelial regeneration in the pleura. No mucociliary clearance in the pleural cavity is a predisposing factor to establish infection in pleura as well. All of above mentioned factors are crucial for pathogenesis of pleural aspergillosis, because *Aspergillus* does not require viable tissue, but prefers necrotic tissue, aerobic conditions and protein-rich exudates. These components may be easily found in the pleural cavity of the present patient. This may explain the observed presence of fungal hyphae in the pleura without involvement of the lung parenchyma.

Notably, reassessment of the preoperative imaging with ^18^F-FDG PET/CT raised further questions regarding the marked pleural thickening with increased glucose uptake. Although pleural thickening is an early manifestation of pulmonary aspergillosis, pleural aspergillosis does not have to be associated with pulmonary aspergillosis [8]. It was considered whether these imaging findings had been incorrectly attributed to prior pleurodesis rather than to pleural aspergillosis.

The diagnosis of *Aspergillus* infection remains challenging; clinicians need a combination of host factors, clinical symptoms, imaging findings, and surrogate laboratory markers, along with microbiological and histological data, to establish a definitive diagnosis [12,18]. In the case of pleural aspergillosis, Zhang et al. established the diagnosis based on pleural tissue culture and biopsy, along with pleural fluid culture [5]; however, despite the initial galactomannan measurement in some of the patients, no follow-up values were obtained to assess the response to treatment. In another case of pleural aspergillosis, the measurement of pleural galactomannan was suggested to monitor the response to treatment [27]. In the case of our patient, the diagnosis was driven by tissue histopathology and culture; the use of serum markers (galactomannan, β-D glucan) was not useful as diagnostic markers or for monitoring.

Microbiological diagnosis established by consecutive fungal culture from normally sterile pleural fluid and histopathological examination of angioinvasion in pleural biopsy specimens are both the gold standard for the diagnosis of invasive pleural aspergillosis. However, these methods are invasive and time-consuming. In many immunocompromised patients, biopsy may be precluded by coexisting conditions such as thrombocytopenia. As a result, antigen detection, especially GM, has become a widely used, less-invasive diagnostic approach in clinical practice.

Galactomannan (GM) is a polysaccharide component of the cell wall of *Aspergillus* spp. that is released into the bloodstream by growing hyphae and germinating spores/conidia [28]. After tissue invasion by *Aspergillus*, GM is released into the blood and other body fluids (BAL, CSF, and pleural fluid) [29]. It has been suggested that the concentration of GM is proportional to the fungal load in tissue and that the level of GM has a prognostic value [28].

There is a commercially available sandwich enzyme-linked immunosorbent assay (ELISA) test for the detection of GM in serum, which can also be applied on other body fluids: bronchoalveolar lavage, cerebrospinal fluid, pericardial fluid and urine, with results reported within 4–6 h [30,31]. Case reports of pleural aspergillosis with GM testing in pleural fluid are rare. During the diagnosis of pleural aspergillosis, the GM identification is generally not performed in pleural fluid, bronchoalveolar lavage fluid or in blood samples [16].

Non-cultured methods, like serum GM testing, have a high diagnostic accuracy for the diagnosis of aspergillosis in patients suspected of having invasive aspergillosis. So far, evidence-based guidelines for the use of GM testing for the diagnosis of pleural aspergillosis have not been published; the guidelines are focused on the use of the GM antigen in invasive pulmonary and disseminated aspergillosis in patients with immunocompromised conditions [32]. The diagnostic yield of *Aspergillus* GM is higher when tested in BAL samples than in serum samples in patients receiving immune suppressants. Due to the high rate of false-positive results at the recommended GM cutoff of 0.5 in serum and BAL, a higher cutoff of 1.0 has been proposed in the revised EORTC-MSGERC criteria. Although GM ELISA shows a good diagnostic accuracy for IA, there are insufficient data to determine its clinical utility, particularly in pleural aspergillosis [28].

The in vivo sGM results in pleural aspergillosis may vary depending on the site and extent of infection. The concentration is determined by the balance between GM production and secretion by actively growing fungal hyphae, but also by the dynamics of its absorption into the bloodstream and its subsequent clearance from the circulation [18]. A positive GM in pleural fluid (pfGM) (elevated at 2.53) with negative sGM was documented in the literature, reflecting localized antigen release within the pleural space without systemic dissemination [33]. Conversely, sGM may be positive while pfGM remains negative, particularly in cases of systemic or pulmonary aspergillosis with limited pleural involvement, but there are no data in the literature to support this assumption. It is also possible for both pfGM and sGM to be positive, indicating both local and systemic antigen presence. However, both compartments may also yield negative GM results despite confirmed pleural aspergillosis, likely due to low fungal burden, limited angioinvasion, or reduced antigen release [15]. Undetectable GM in this study may indicate low antigen levels, suggesting that GM detection depends on fungal burden and location of *Aspergillus* infection. Despite a negative GM test, a patient can still be classified as having ‘probable’ pleural aspergillosis (based on another microbiological criteria) or as having ‘proven’ pleural aspergillosis based on microbiological culture/histopathological findings) [34]. Therefore, GM is considered a supportive biomarker in at-risk patients and GM results must be interpreted in conjunction with clinical, microbiological and radiological data [34]. GM results in pleural fluid do not have an established diagnostic threshold, and their sensitivity and specificity in this location are not well studied, so caution is still advised in clinical interpretation.

Given the rarity of pleural aspergillosis cases reported in the literature, the sparsity of data to guide management is not surprising. Zhang et al. reported the combination of systemic voriconazole, local lavage with amphotericin B solution, and thoracic drainage [5]. A daily local administration of amphotericin B (25 mg in 500 mL of 5% glucose solution) was applied with closed thoracic drainage. However, this is not the first report of topical administration of antifungals for pleural *Aspergillus* infections. A young adult submitted to pleurodesis for recurrent pneumothorax was treated for pleural aspergillosis with a systematic antifungal (initially voriconazole, which was switched to liposomal amphotericin B due to liver toxicity) combined with local deoxycholate amphotericin B administration through the chest drainage. Specifically, 5 mL of conventional amphotericin B diluted in 70 mL of 5% dextrose was administered via the thoracic tube [16]. The local administration was discontinued a few days later due to concerns about a new febrile episode that was later attributed to pyelonephritis [16]. In another case, a 54-year-old male previously submitted to lobectomy due to lung cancer developed a BPF after surgery and was further managed with open thoracostomy; the pleural culture showed *Streptococcus pneumoniae* [27]. His condition was further complicated by *Aspergillus fumigatus* isolation in tissue biopsy, which was treated with systemic voriconazole (sub-therapeutic levels) combined with topical liposomal amphotericin B applied with gauzes via the thoracostomy [27]. In this case, 50 mg of liposomal amphotericin B within 100 mL of 5% dextrose was implemented on gauze, locally [27]. In another case, 200 mg of reconstituted amphotericin B deoxycholate with 4 g of hemostat powder was administered for fungal empyema and BPF as a supplementary treatment to the systemic antifungal agent [35]. In an elderly patient with interstitial pneumonia related to systemic sclerosis, who was operated on due to a cavitary lesion attributed to both nontuberculous mycobacteria and *Aspergillus* spp., systemic and local antifungal agents were combined after open-window thoracostomy [36]. The local application of amphotericin B on gauzes infused within 50 mL normal saline was gradually increased from 1 mg (day 1) to 5 mg (day 3), to 10 mg (day 8), up to a maximum dose of 25 mg (day 16), and for a total 2-month course after operation, with successful management of infection [36]. Intrapleural administration of liposomal amphotericin B alongside the systemic antifungal regimen has also been described in a pediatric patient [37].

Evidence from a retrospective analysis of 36 patients treated with both systemic and local drugs (nebulizer or intrapleural or bronchoscopic administration) for fungal infections showed that topical implementation was well tolerated and safe [38]. These data, however, should be interpreted with caution because most of the patients received inhaled antifungals and only two were treated with intrapleural administration [38]. Finally, the bioavailability of the systemic antifungal agent in the thickened pleura may be doubtful; a low intrapleural concentration of liposomal amphotericin B would require longer courses of the drug at high doses [39,40].

Despite the limited literature, clinicians may consider intrapleural instillation of antifungal agents. First, adjunctive local therapy may be considered when a primary systemic antifungal agent, such as voriconazole, cannot reach therapeutic concentrations due to either limited bioavailability in the pleura or due to impaired pharmacokinetics related to host factors. Intrapleural antifungal instillation may also be considered when systemic therapy poses a risk of toxicity due to dose escalation required to achieve the therapeutic drug concentrations or due to other adverse effects.

## 4. Conclusions

Pleural aspergillosis is a rare extrapulmonary manifestation of invasive *Aspergillus* infection, associated with thoracic surgical procedures and with a bronchopleural fistula, not necessarily combined with invasive pulmonary aspergillosis, primarily in individuals with aggressive immunosuppression, but also in those without immunosuppression. This case presents the postoperative isolated form of pleural aspergillosis, established by culture-confirmed fungal growth and histopathological identification of hyphae, both from pleural specimens, in an immunosuppressed patient with sarcoma. Extended thoracoplastic procedures are often unavoidable to achieve effective control in pleural aspergillosis, as well as being essential for survival despite their disfiguring and mutilating effects. The pleural aspergillosis and its potential complications require a combination of conventional surgical approaches with both systemic and local antifungal treatment regimens. As reported in this study, intrapleural administration of an antifungal agent through a thoracostomy may represent a rational therapeutic option when systemic antifungal toxicity, uncertain intrapleural pharmacokinetics and subtherapeutic intrapleural concentrations limit effective therapy. Postoperative extrapulmonary, invasive aspergillosis with pleural involvement and bronchopleural fistula, in immunocompromised patients, is rarely reported; therefore, this case contributes to clinical experience. Current pleural aspergillosis management is not standardized and is still based on documented individual case data, emphasizing the need for continued case reporting to refine evidence-based approaches.

## Data Availability

The data presented in this study are available in this article.

## Abbreviations

The following abbreviations are used in this manuscript:

ABPA	allergic bronchopulmonary aspergillosis
BPF	bronchopleural fistula
COPD	chronic obstructive pulmonary disease
CPA	chronic pulmonary aspergillosis
CT	computed tomography
IA	invasive aspergillosis
IPA	invasive pulmonary
